# Apocynin Mitigates Diabetic Muscle Atrophy by Lowering Muscle Triglycerides and Oxidative Stress

**DOI:** 10.3390/ijms26125636

**Published:** 2025-06-12

**Authors:** Sarai Sánchez-Duarte, Elizabeth Sánchez-Duarte, Luis A. Sánchez-Briones, Esperanza Meléndez-Herrera, Ma. Antonia Herrera-Vargas, Sergio Márquez-Gamiño, Karla S. Vera-Delgado, Rocío Montoya-Pérez

**Affiliations:** 1 Instituto de Investigaciones Químico Biológicas, Universidad Michoacana de San Nicolás de Hidalgo, Francisco J. Mújica S/N, Col. Felicitas del Río, Morelia 58030, Michoacán, Mexico; 1315649c@umich.mx; 2 Departamento de Ciencias Aplicadas al Trabajo, Universidad de Guanajuato Campus León, Eugenio Garza Sada 572, Lomas del Campestre Sección 2, León 37150, Guanajuato, Mexico; elizabeth.sanchez@ugto.mx (E.S.-D.); luis.sanchezb@ugto.mx (L.A.S.-B.); smgamino@fisica.ugto.mx (S.M.-G.); ksvera@ugto.mx (K.S.V.-D.); 3 Instituto de Investigaciones Sobre los Recursos Naturales, Universidad Michoacana de San Nicolás de Hidalgo, Ave. Juanito Itzícuaro SN, Morelia 58330, Michoacan, Mexico; emelendez@umich.mx (E.M.-H.); antonia.herrera@umich.mx (M.A.H.-V.)

**Keywords:** diabetes, atrophy, skeletal muscle, triglycerides, apocynin, oxidative stress

## Abstract

Diabetic muscular atrophy is a complication of diabetes mellitus that can decrease quality of life. Its complex mechanisms include alterations in proteolytic pathways, oxidative stress, and intracellular lipid accumulation. NADPH oxidase enzymes (NOX) play a key role in the production of ROS, contributing to oxidative damage and insulin resistance. Apocynin, a NOX inhibitor, has antioxidant and anti-inflammatory effects, suggesting its therapeutic potential in various diabetic complications. This study evaluated the impact of apocynin on the mechanisms of muscle atrophy in slow- and fast-twitch muscles of diabetic rats. Diabetes was induced in male Wistar rats by intraperitoneal injection of a single dose of streptozotocin (60 mg/kg). Apocynin treatment (3 mg/kg/day) was administered for 8 weeks. Fasting blood glucose levels, lipid profile, and weight gain were measured. Both slow-twitch (soleus) and fast-twitch (extensor digitorum longus, EDL) skeletal muscles were weighed and used to assess triglycerides (TG) content, histological analysis, lipid peroxidation levels, and gene expression evaluated by qRT-PCR. Apocynin reduced blood glucose levels, improved body weight, and exhibited hypolipidemic effects. It significantly increased muscle weight in EDL and soleus, especially in EDL muscle, lowering triglycerides, lipid peroxidation, and increasing fiber size. Additionally, it decreased mRNA expression levels of MuRF-1, atrogin-1, myostatin and p47phox mRNA and upregulated PGC-1α and follistatin mRNA. Apocynin exerted a myoprotective effect by mitigating muscle atrophy in diabetic rats. Its effects were differentially mediated on TG accumulation and muscle fiber size, reducing oxidative stress, atrogene expression, and positively regulating PGC-1α.

## 1. Introduction

Diabetes mellitus (DM) is a chronic metabolic disease characterized by elevated blood glucose levels, which, over time, disrupt glucose, lipid, and protein metabolism due to either insulin insufficiency (type 1) or insulin resistance (type 2) [1,2]. DM induces muscle atrophy and a transition from oxidative to glycolytic muscle fibers, reducing the oxidative capacity in skeletal muscles [3,4,5]. This impacts muscle development, worsening diabetic myopathy, muscle dysfunction, weakness, and increased exercise intolerance [6]. Increased protein degradation and reduced synthesis have been observed in muscles of streptozotocin (STZ)-induced diabetic rats and in DM patients [7]. Atrophy, characterized by the decrease in muscle size and strength, involves proteolytic pathways such as the ubiquitin–proteasome system and autophagy–lysosome pathway primarily [8]. Factors such as hyperglycemia, elevated glucocorticoids, inflammation, insulin resistance, oxidative stress, and dyslipidemia contribute to the development of diabetic muscle atrophy [9].

The increase in intramyocellular lipids (IMCLs), particularly triglycerides, has been associated with the development of insulin resistance in patients with type 1 diabetes and animal models [10]. Although IMCLs are essential for energy metabolism, their ectopic storage in skeletal muscle, resulting from insulin resistance in peripheral tissues, leads to the accumulation of lipotoxic intermediates such as diacylglycerols (DAGs) and ceramides. These, in turn, driven by insulin resistance, can create a feedback loop that damages muscle function and potentially leads to loss of muscle mass. Palmitate in myotubes has been reported to induce ceramide accumulation, increasing the expression of proatrophic genes such as atrogin-1/MAFbx, elevating FoxO3, and reducing protein synthesis [11].

Oxidative stress is a central factor linked to the development of diabetic complications. Hyperglycemia promotes the overproduction of reactive oxygen species (ROS), leading to redox imbalance and cellular damage. Specifically, in skeletal muscle, high levels of ROS wreak havoc within the tissue, disrupting insulin signaling, promoting lipotoxicity and mitochondrial dysfunction, and activating inflammatory pathways, thereby contributing to muscle dysfunction and atrophy [12,13], as well as modifying the expression of genes involved in the regulation of muscle mass and oxidative capacity [14]. In skeletal muscle, while mitochondria also contribute to ROS production, nicotinamide adenine dinucleotide phosphate (NADPH) oxidases (NOX) enzymes, localized to the sarcolemma, transverse tubules, and sarcoplasmic reticulum, are a major source of cytosolic ROS, especially during muscle contraction in health and disease [15,16]. The NOX enzyme family consists of seven members, and two isoforms (NOX2 and NOX4) are present in skeletal muscle [16]; an increase in the activity and gene expression of NOX and certain members of the complex have been implicated in various aspects of diabetes-related metabolic dysregulation, including insulin resistance and glucose transport [17,18,19]. It has been reported that their activity and expression are regulated by angiotensin II (AT II), growth factors, mechanical stress/contraction, and high glucose, insulin, and fatty acid levels [12]. The involvement of NOX2 in muscle atrophy induced by disuse or denervation in animal models and Duchenne muscular dystrophy in mdx mice has been established [20]. The involvement of NOX2 in muscle atrophy models induced by AT II has also been reported [21]. Therefore, NOX2 inhibition is a potential therapeutic strategy; however, its role in diabetic muscle atrophy has yet to be elucidated.

Apocynin is a drug isolated from the root extracts of the medicinal herb Picrorhiza kurroa [22]. Various studies in animal and cellular models have shown that it is an effective inhibitor of reactive oxygen species (ROS) generation by NOX enzymes [23,24,25]. This compound has been widely used in experimental studies, demonstrating therapeutic potential in various pathologies and diabetic complications due to its antioxidant and anti-inflammatory properties [22]. It has been found that apocynin significantly reduces hyperglycemia, hyperinsulinemia, and insulin resistance in high-fat-diet-induced obese mice [26], in addition to restoring the activity of serum antioxidant enzymes such as catalase and superoxide dismutase (SOD) in diabetic rats [27]. Moreover, apocynin has been reported to improve insulin sensitivity, attenuate oxidative stress, and preserve mitochondrial function in the cardiac muscle of diabetic rats [25]. In skeletal muscle, we previously demonstrated the myoprotective effects of apocynin against oxidative stress, attenuating muscle dysfunction induced by diabetes in both fast and slow muscles through the regulation of the Nuclear Factor-kappa B (NF-κB) and Nrf2 pathways [28]. However, to date, the effect of apocynin on diabetic muscle atrophy associated with lipid metabolism alterations has not been investigated. It is essential to highlight that diabetic muscle atrophy is a less explored complication, and it can significantly reduce the quality of life. Given the previously indicated background, we hypothesized that apocynin could protect against diabetic muscular atrophy and concomitant hyperlipidemia. This study aimed to evaluate the impact of apocynin on muscle mass, muscle lipid content, lipid peroxidation, fiber size, the expression of genes involved in regulating muscle mass using a rat model of STZ-induced diabetes.

## 2. Results

### 2.1. Effect of Apocynin on Blood Glucose and Lipid Profile

**Figure 1:**
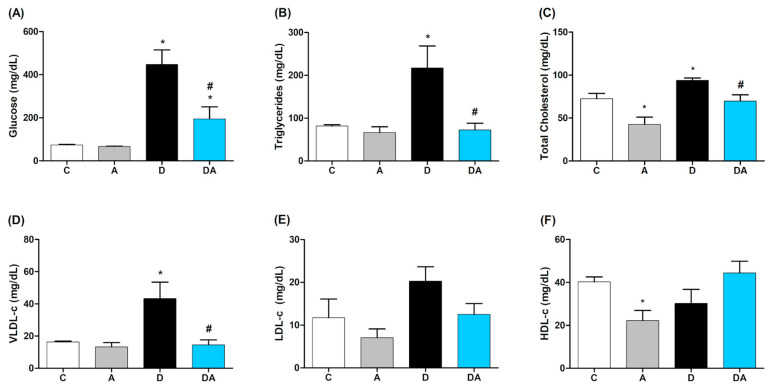
Effect of apocynin on metabolic biomarkers. (**A**) Blood glucose, (**B**) triglycerides, (**C**) cholesterol, (**D**) VLDL-c: lipoprotein low density, (**E**) LDL-c: very low-density lipoprotein, and (**F**) HDL-c: high-density lipoproteins. C = control group; A = apocynin group; D = diabetic group; DA = diabetes + apocynin group. Data are presented as the mean ± SEM (*n* = 8 per group). * *p* < 0.05 vs. C group. # *p* < 0.05 vs. D group.

The impact of NOX inhibition with apocynin on fasting blood glucose levels was analyzed (Figure 1A). The diabetic group showed elevated glucose levels (447 ± 68.2 mg/dL) compared to the control group (73.5 ± 2.96 mg/dL; *p* = 0.0002), confirming the experimental DM. However, the diabetic group treated with apocynin showed a significant reduction (194.6 ± 56.25 mg/dL; *p* = 0.0101) compared to the untreated diabetic group. The lipid profile is shown in Figure 1B–F. Compared to the control group, the triglycerides, total cholesterol, and VLDL levels were significantly elevated in the diabetic group (*p* ≤ 0.05), indicating the presence of diabetic dyslipidemia. The administration of apocynin under the same conditions significantly prevented this increase, showing a similar trend to control group in triglycerides (*p* = 0.0070) (Figure 1B), total cholesterol (*p* = 0.0301) (Figure 1C), and VLDL (*p* = 0.0070) (Figure 1D) levels. No significant changes were observed in LDL (Figure 1E) and HDL (Figure 1D) levels compared to the diabetic group. These results suggest a potential hypolipidemic effect of apocynin.

### 2.2. Effect of Apocynin on Body Weight and Muscle Mass

**Figure 2:**
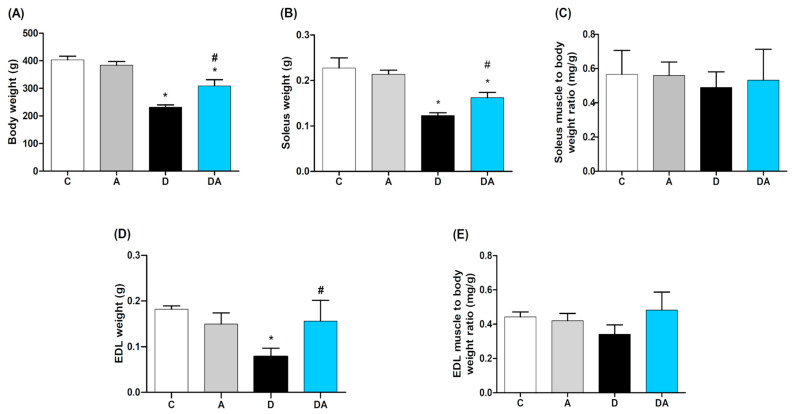
Effect of apocynin on body weight (**A**), soleus muscle weight (**B**), normalized soleus muscle weight to body weight (**C**), EDL muscle weight (**D**), EDL muscle weight to body weight ratio (**E**). C = control group; A = apocynin group; D = diabetic group; DA = diabetes + apocynin group. Data are presented as the mean ± SEM (*n* = 8 per group). * *p* < 0.05 vs. C group. # *p* < 0.05 vs. D group.

Regarding body weight (Figure 2A), the diabetic group exhibited a significant weight loss (231.7 ± 8.586 g) compared to the control group (391.8 ± 10.68 g; *p* = 0.0001). This effect was reversed with apocynin, showing a significant improvement in body weight (309.0 ± 22.38 g; *p* = 0.0050) compared to the untreated diabetic group. The apocynin group did not show significant changes in these parameters. The muscle weight was significantly reduced in both muscles of diabetic rats, specifically in the soleus (Figure 2B) and EDL (Figure 2C) (*p* = 0.0002; *p* = 0.0001, respectively). Apocynin increased muscle weight in diabetic rats by 51.3% in the EDL (*p* = 0.0300) and 78.2% in the soleus (*p* = 0.0210) compared to the diabetic group. No statistically significant changes were observed in the muscle weight-to-body weight ratio among the experimental groups for both muscles Furthermore, the muscle weight/body weight ratio was measured to assess muscle mass relative to overall body weight. It was calculated by dividing the weight of a spe-cific muscle by the total body weight. Consequently, no statistically significant changes were observed in the muscle weight-to-body weight ratio among the experimental groups for both muscles (Figure 2C,E).

### 2.3. Effect of Apocynin on the Size and Number of Skeletal Muscle Fibers in Diabetic Rats

**Figure 3:**
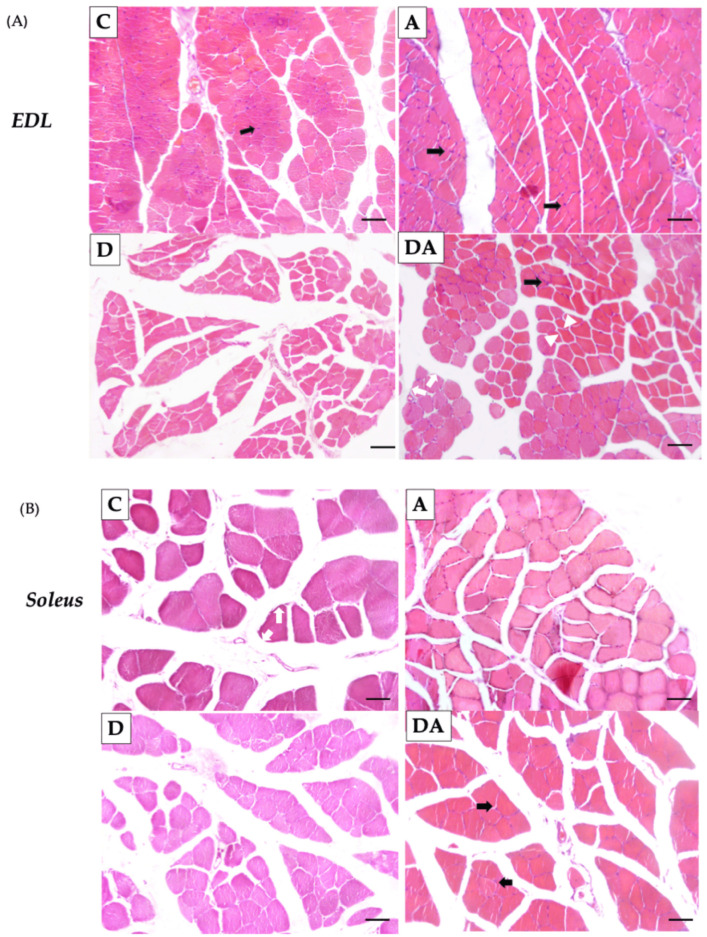

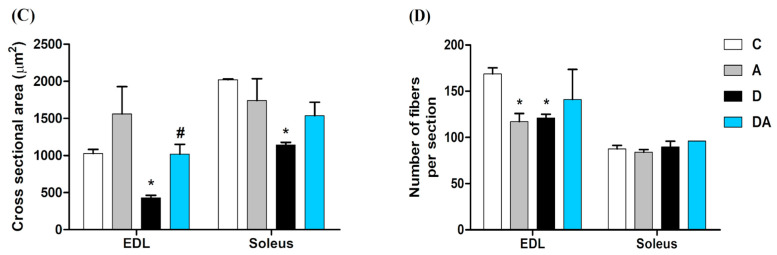
Effect of apocynin on muscle morphology. Morphological changes were observed in the EDL muscle (**A**) and the soleus muscle (**B**), scale bar, 50 µm. Muscle fibers (stained pink) exhibit homogeneous sizes within each experimental group. Connective tissue is observed in the perimysium (white arrows) and endomysium (white arrowhead). Nuclei (dark purple staining) are located at the periphery of the muscle fibers (black arrows). Cross-sectional area (μm^2^) (**C**) and number of fibers per section (**D**). C = control group; A = apocynin group; D = diabetic group; DA = diabetes + apocynin group. Data are presented as mean ± standard error of the mean (SEM) (*n* = 8 per group). * *p* < 0.05 vs. C group. # *p* < 0.05 vs. D group.

Histological analysis revealed the number of fibers and the cross-sectional area (CSA) in the EDL (Figure 3A) and soleus muscles (Figure 3B). It was observed that the number of fibers per section was lower in the diabetic group compared to the control group in the EDL muscle, but no significant changes were observed in the soleus muscle compared to all groups (Figure 3C). Additionally, the CSA was significantly reduced in the diabetic group in both muscles compared to the control group (*p* = 0.0008 in EDL muscle; *p* = 0.0004 in the soleus muscle) (Figure 3D). In contrast, treatment with apocynin increased the CSA in EDL muscle compared to the diabetic group (*p* = 0.0112), while no significant changes were observed in the soleus muscle (Figure 3C). However, apocynin did not affect the number of fibers per section in the muscles of diabetic rats (Figure 3D).

### 2.4. Inhibition of Nox by Apocynin Reduces Muscle Triglycerides Content in Diabetic Rats

**Figure 4:**
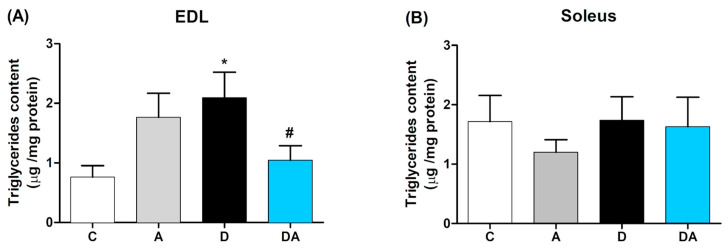
Effect of apocynin on intramuscular triglycerides content of diabetic rats in both EDL (**A**) and soleus (**B**) muscles. Apocynin significantly reduced the content of intramuscular triglycerides in the EDL muscle of diabetic rats. C = control group; A = apocynin group; D = diabetic group; DA = diabetes + apocynin group. Data are presented as the mean ± SEM (*n* = 6 per group). * *p* < 0.05 vs. C group. # *p* < 0.05 vs. D group.

The triglycerides content in the skeletal muscles of diabetic rats was determined, showing significant differences between slow-twitch (soleus) and fast-twitch (EDL) muscles (Figure 4). In the EDL muscle (Figure 4A), the triglycerides content was higher in the diabetic group compared to the control group (*p* < 0.05). However, treatment with apocynin for eight weeks reduced this content in the diabetic group (*p* < 0.05). In the soleus muscle (Figure 4B), no significant differences were observed among the four groups. 

### 2.5. Apocynin Reduces Lipid Peroxidation in Fast and Slow Diabetic Skeletal Muscles

**Figure 5:**
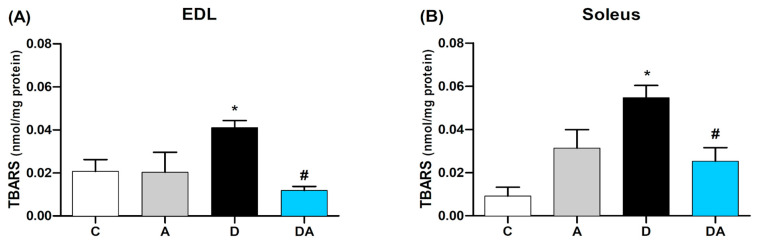
Effect of apocynin on thiobarbituric acid reactive substances (TBARS) levels in (**A**) EDL and (**B**) Soleus. Data are presented as mean ± SEM. C = control group; A = apocynin group; D = diabetic group; DA = diabetes + apocynin group. (*n* = 8). * *p* < 0.05 vs. C group. # *p* < 0.05 vs. D group.

We determined TBARS levels as an indicator of lipid peroxidation in the skeletal muscles of diabetic rats as part of the assessment of oxidative stress. As shown in Figure 4, a significant increase in TBARS concentration was observed in the EDL (Figure 5A) and soleus (Figure 5B) muscles of diabetic rats compared to the control group (*p* = 0.0020). The treatment with apocynin prevented the increase in lipid peroxidation in the EDL muscles by 28.8% and in the soleus by 46.1% (*p* = 0.0001 and *p* = 0.0116, respectively) in the diabetic + apocynin group, compared to the diabetic group. In contrast, apocynin did not significantly alter TBARS levels in the EDL and soleus muscles of the control group.

### 2.6. Effect of Apocynin on the Gene Expression of Muscle Mass Regulators and Oxidative Capacity in the Skeletal Muscle of Diabetic Rats

**Figure 6:**
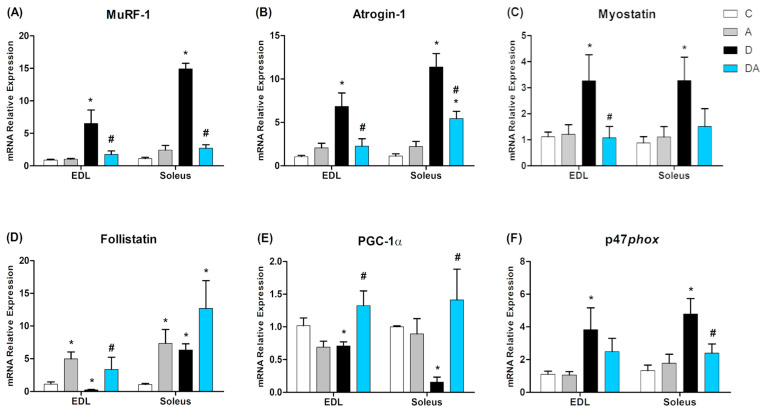
Effect of apocynin on mRNA expression levels of *MuRF-1* (**A**), *Atrogin-1* (**B**), *myostatin* (**C**), *follistatin* (**D**), *PGC-1α* (**E**), and *p47phox* (**F**) in both EDL and soleus muscles. C = control group; A = apocynin group; D = diabetic group; DA = diabetes + apocynin group. Data are presented as the mean ± SEM (*n* = 8 per group). * *p* < 0.05 vs. C group. # *p* < 0.05 vs. D group.

Gene expression changes were analyzed using RT-qPCR in fast-twitch (EDL) and slow-twitch (soleus) skeletal muscles, as shown in Figure 6. In the diabetic group, an increase in the expression of *MuRF-1* (Figure 6A) and *Atrogin-1* (Figure 6B) were significantly higher in both muscles compared to the control group. *MuRF-1* levels were significantly higher in EDL (*p* = 0.0221) and soleus (*p* = 0.0001) and for *Atrogin-1* in EDL (*p* = 0.0042) and soleus (*p* = 0.0062). Apocynin treatment downregulated the expression of both atrogenes, with a 24.6% decrease in *MuRF-1* in EDL (*p* = 0.0001) and an 18.6% decrease in soleus (*p* = 0.0001) and a 33.1% reduction in *Atrogin-1* in EDL (*p* = 0.0336) and a 47.7% reduction in soleus (*p* = 0.0062), compared to the diabetic group. 

In Figure 6C, *myostatin* mRNA expression was upregulated in the diabetic group in both EDL (33.6%; *p* = 0.0335) and soleus muscle (32.9%; *p* = 0.0112). NOX2 inhibition by apocynin significantly reduced *myostatin* levels in EDL (*p* < 0.05; 43.3%) compared to the diabetic group. Regarding *follistatin* (Figure 6D), elevated expression was observed in the apocynin group (*p* = 0.0038; 23%) compared to the control. In the diabetic + apocynin group, a significant reduction was recorded in EDL (*p* = 0.0271; 22%) but not in soleus.

Changes in *PGC-1α* mRNA levels were also analyzed (Figure 6E). In the diabetic group, a significant reduction was observed in both EDL (50.3%; *p* = 0.0033) and soleus muscles (47%; *p* = 0.0002). Apocynin treatment significantly increased *PGC-1α* expression in EDL (33.3%; *p* = 0.0210) and soleus (11.1%; *p* = 0.0445). Finally, *p47phox* mRNA expression (Figure 6F) was prominently increased in the diabetic group in both muscles compared to those from the control group (*p* < 0.05). Apocynin treatment reduced *p47phox* expression in the soleus, decreasing it by 49.9% compared to the diabetic group (*p* = 0.0305), but there was no difference in expression in the EDL muscle when compared to all groups.

## 3. Discussion

Diabetic myopathy, a muscle disorder associated with diabetes, is characterized by disturbances in glucose, lipid, and protein metabolism, which compromise the structural integrity and functional capacity of skeletal muscle and potentially contribute to other diabetic complications [4]. Several studies have highlighted that effective diabetes management should focus on improving glycemic control, addressing abnormalities in lipid metabolism, and mitigating oxidative stress [29,30,31]. In the present study, we evaluated the effects of apocynin, a potent inhibitor of NADPH oxidase with antioxidant properties, on the mechanisms of muscle atrophy induced by diabetes and concomitant hyperlipidemia. Our findings for the first time demonstrate that apocynin can attenuate diabetes-induced skeletal muscle atrophy. This protective effect was associated with the downregulation of genes linked to muscle atrophy, such as *Atrogin-1*, *MuRF-1*, and *myostatin*, as well as the cytosolic subunit of NOX, *p47phox*. Concurrently, we observed an upregulation of factors involved in muscle homeostasis, such as *follistatin* and *PGC-1α*. In addition, apocynin reduced the susceptibility of skeletal muscle to lipid peroxidation and demonstrated hypolipidemic effects.

Our STZ-induced diabetic model evidenced an increase in glucose levels and a significant reduction in body weight at the end of the experimental protocol. STZ induces the destruction of pancreatic β-cells, leading to a hyperglycemic state due to the ablation of insulin [32]. Insulin deficiency favors protein degradation to supply the amino acids required for gluconeogenesis, which explains the observed loss of muscle mass and decreased body weight [33]. Under similar conditions, the administration of apocynin for eight weeks (3 mg/kg) exhibited hypoglycemic properties and improved weight gain [28], which is consistent with our results and further reinforces the anti-diabetic properties of apocynin [28,33].

Loss of muscle mass is a key predictor of muscle atrophy [4]. In diabetes, insulin deficiency and prolonged hyperglycemia result in muscle wasting, altered metabolic capacity, and reduced muscle function [34]. Previously, it was observed that administering apocynin for five and eight weeks improved insulin sensitivity in diabetic rats [25,28] and enhanced muscle function [28], thereby contributing to muscle health. The results of the present study demonstrated that apocynin treatment increased muscle mass; we observed an increase in muscle weight in both EDL and soleus muscles in diabetic rats. The EDL is typically classified as a fast-twitch muscle due to its myosin isoform expression profile, whereas the soleus is considered a slow-twitch muscle [35]. Both muscles are commonly used to evaluate the muscle-to-body weight ratio [36]. Following apocynin administration, the increase in this ratio suggests a possible stimulation of protein synthesis or inhibition of its accelerated degradation processes frequently impaired under diabetic conditions [4]. Moreover, a previous study reported that apocynin treatment preserved muscle mass and function in a smoking-induced atrophy model by maintaining the proteostatic pathway [37], which is consistent with our findings.

Diabetic myopathy, by affecting skeletal muscle, increases insulin resistance and compromises its ability to regulate blood glucose and lipid profile [38]. In our study, we observed diabetic dyslipidemia, characterized by elevated levels of triglycerides (TG), total cholesterol, and VLDL-c, consistent with previous findings in diabetic rat models [39,40]. Interestingly, apocynin attenuated lipid alteration at a systemic level in diabetic rats, showing a hypolipidemic effect. This effect could be related to its ability to reduce dyslipidemia and insulin resistance and exert a hepatoprotective effect by mitigating oxidative stress in obese mice [26]. Furthermore, previous studies have shown that apocynin reduces inflammation in adipose tissue by modulating the altered activity of key enzymes involved in lipid metabolism [26,41,42].

Metabolic rigidity in the liver and adipose tissue contributes to the ectopic accumulation of lipids in non-adipose tissues, such as skeletal muscle, which is associated with lipotoxicity [43]. Although TG are not direct lipotoxic species, they may reflect the overall lipid load [34,44], and evidence indicates that excessive storage of TG within skeletal muscle is linked to insulin resistance [45]. In our study, we found high TG levels in the EDL muscle of the diabetic group, as has been confirmed in other studies conducted on skeletal muscles under this condition [46,47]. However, TG levels did not change in the diabetic soleus muscle in comparison to the control group; this suggests that diabetes-related lipid buildup affects in a muscle-type-specific manner. Similarly, studies by Umek et al. [48] showed that fast-twitch muscles are more susceptible to lipid accumulation compared to slow-twitch muscles, such as the soleus in obese mice. This difference is linked to their metabolic characteristics and susceptibility to metabolic stress, which in turn affects the rate of lipid storage and the ability to clear circulating lipids [48]. In line with this fact, studies have shown that intramuscular fat accumulation (LIMC) is associated with loss of strength and muscle atrophy [4,34,49]. Remarkably, apocynin prevented these alterations; it decreased the accumulation of TG in the EDL muscle but did not influence the soleus muscle. These findings indicate that apocynin has differential impacts on skeletal muscle lipid metabolism. Despite these results, previous studies on the protective effects of apocynin on both muscles have shown that its action is predominantly due to its antioxidant properties [28]. Specifically, apocynin mitigated oxidative stress by decreasing ROS levels and increasing total glutathione levels and redox state in both types of muscles [28]. In line with this, the current study showed that apocynin significantly reduced TBARS levels in both muscles, indicating decreased lipid peroxidation. Lipid peroxidation is a damaging characteristic of oxidative stress that results in the oxidative destruction of lipids, which damages cellular membranes, affects metabolic processes and cell signaling [50], and is associated with insulin resistance in muscle fibers [51]. Similarly, apocynin also reduced lipid peroxidation in the liver tissue of mice fed a high-fat diet [26,42]. Lower TBARS levels may help prevent oxidative stress-induced skeletal muscle damage [52], which could lessen muscular atrophy and enhance muscle function [53].

The findings of this study suggest that apocynin treatment attenuates oxidative stress in both types of skeletal muscle evaluated. However, its effects differed regarding muscle mass preservation, TG content, and muscle CSA. These results are consistent with previous studies indicating that oxidative stress more severely affects muscles predominantly composed of fast-twitch fibers than those with a predominance of slow-twitch fibers [54], which aligns with the morphological alterations observed in the present work. Notably, although apocynin induced a significant increase in CSA only in the EDL muscle, it did not influence the number of fibers, even though diabetes is associated with a reduction in this cell population, a phenomenon also evidenced in the EDL. Based on previous research by our group, these results could be linked to a functional protective effect potentially linked to the modulation of oxidative stress [28]. Diabetes-induced redox imbalance has been widely associated with structural and functional alterations in skeletal muscle, negatively impacting its metabolic capacity. In this regard, elevated levels of oxidative damage markers have been associated with a reduction in muscle fiber size and impaired contractile strength, contributing to the development of muscle fatigue [55]. Therefore, our results further support the role of oxidative stress as a key factor in diabetic myopathy and suggest that apocynin may counteract these adverse effects.

This study proposes that oxidative stress mediated by NOX2 contributes to insulin resistance and skeletal muscle atrophy. It has been shown that skeletal muscle expresses two NOX isoforms, NOX2 and NOX4, which are localized in the sarcolemma, sarcoplasmic reticulum, and T-tubules of muscle fibers. Furthermore, NOX activity and expression vary depending on the skeletal muscle fiber type and the tissue antioxidant capacity [28,56]. Several studies have associated dysregulated NOX2 activity with functional and structural alterations of skeletal muscle under pathological conditions [57]. A previous study demonstrated that NOX2 and NOX4 are involved in diabetes-induced muscle dysfunction [28]. In addition, metabolic factors such as hyperglycemia and lipid overload have been reported to modulate NOX2 expression and activity [12]. On the other hand, Bechara et al. [58] showed that muscle atrophy in a heart failure model is associated with increased NOX2 mRNA expression, suggesting that this isoform may be primarily responsible for the heightened NOX activity observed in various pathological states of skeletal muscle. We observed a significant increase in the expression of the atrogenes *Atrogin-1* and *MuRF-1* in the soleus and EDL muscles of diabetic rats, genes commonly associated with muscle atrophy in STZ-induced experimental models of diabetes [11,59]. Inhibition of NOX2 by apocynin downregulates these genes in both muscles, suggesting a protective effect against protein degradation. These findings are consistent with those reported by Chan et al. [37] in a model of muscle atrophy induced by chronic obstructive pulmonary disease (COPD) following apocynin administration. This compound has been reported to act as a ROS scavenger [60], which could underlie the effects observed in our study. Elevated levels of ROS promote protein degradation and inhibit protein synthesis, contributing to muscle mass loss and metabolic dysfunction [61,62]. These findings suggest that NOX2 may be involved in activating proteolytic pathways, possibly through redox imbalance, which agrees with the results reported by Lawler et al. [61].

Muscle atrophy is associated with two main protein degradation pathways: the ubiquitin–proteasome system and the autophagy–lysosome pathway. Additionally, this process is negatively regulated by pathways involved in protein synthesis, such as the IGF-1–PI3K–Akt/mTOR and IGF-1–Akt–FoxO signaling pathways [8,63,64]. Due to its high metabolic activity, skeletal muscle is particularly susceptible to oxidative stress, which plays a key role in the progression of muscle atrophy [65]. ROS act as key mediators in activating pro-inflammatory pathways, such as the NF-κB pathway, which promotes myostatin expression in muscle cells. Myostatin is a negative regulator of skeletal muscle growth and development, as it limits muscle fibers’ number and size. Its expression is increased in STZ-induced diabetes models, consistent with our findings in soleus and EDL muscles [66,67,68]. Interestingly, apocynin treatment downregulated *myostatin* expression, particularly in the EDL muscle, suggesting a differential effect on fast-twitch fibers. We also evaluated the expression of follistatin, a myokine that antagonizes myostatin signaling by preventing its binding to activin type IIB receptors and subsequently inhibiting Smad2/3 pathway activation, thereby suppressing the transcription of *Atrogin-1* and *MuRF-1* [69]. In this study, apocynin treatment led to an upregulation of *follistatin* expression in the EDL muscle. These findings align with those reported by Chan et al. [37], who showed that apocynin reduced *myostatin* expression in myofibers exposed to H_2_O_2_ in a COPD-induced muscle atrophy model. This effect may be mediated, at least in part, by the inhibition of the NF-κB pathway, whose transcriptional activation has been associated with atrophy in fast-twitch fibers and degeneration in slow-twitch fibers [70]. Previous studies have also shown that apocynin reduces *NF-κB* mRNA expression in diabetic rats [28], which may be related to the observed effects on *myostatin* expression.

PGC-1α is a key transcriptional coactivator that protects muscle fibers from atrophy by regulating fiber phenotype, promoting oxidative metabolism, enhancing mitochondrial content, and preserving muscle mass [71,72]. An important finding was the increased expression of *PGC-1α* in the muscles of diabetic rats treated with apocynin. In many biological contexts, particularly in skeletal muscle, upregulation of *PGC-1α* mRNA is indeed associated with parallel increases in PGC-1α protein expression [73]. PGC-1α has been shown to suppress the transcription of *Atrogin-1* and *MuRF-1* through modulation of FoxO, and ROS have been suggested to play a role in its regulation [74]. Furthermore, PGC-1α enhances glucose and lipid transport and oxidation and prevents muscle lipotoxicity, which may correlate with the reduced TG content observed in this study [71]. It has been reported that muscle fibers differ in their susceptibility to atrophy. Oxidative (slow-twitch) fibers are more resistant to degradation, mainly due to PGC-1α activity, whereas glycolytic (fast-twitch) fibers are more vulnerable, particularly in response to stimuli such as FoxO, TGF-β, autophagy inhibition, and NF-κB activation [74,75,76]. This pattern may explain the differential response observed in the EDL muscle following apocynin treatment. 

Additionally, our results demonstrate the transcriptional effects of apocynin on NOX regulation. It has been reported that apocynin inhibits the translocation of the p47phox subunit required for NOX2 activation to the plasma membrane [19,20,21]. In this context, we observed an increase in *p47phox* expression in the muscles of diabetic rats, which was attenuated by apocynin, particularly in the soleus muscle. This result is consistent with previous studies in muscle atrophy models, in which elevated *p47phox* expression was reported along with a reduction in SOD1 and catalase levels [77]. Furthermore, in *p47phox* knockout mice, increased ROS levels and NOX2-dependent muscle atrophy induced by angiotensin II (AII) have been observed [78]. Therefore, these findings suggest, for the first time, that apocynin mitigates muscle at-rophy in diabetes through mechanisms involving NOX2. 

A limitation of this research is the absence of protein-level studies that complement the gene expression findings. We acknowledge that such analyses should be included in future studies to fully delineate the protective effects of apocynin on diabetes-induced structural, functional, and metabolic alterations in skeletal muscle. Nonetheless, we think the results of the study validate the goals of this investigation and advance our knowledge of apocynin as a potentially effective treatment approach to counteract diabetes-induced muscle alterations.

## 4. Materials and Methods

### 4.1. Animal Experiment and Ethical Approval

Male Wistar rats weighing 230–250 g were selected for this study. All animals were housed in a specific pathogen-free environment, with a 12 h light–dark cycle, 22 ± 2 °C. Animals were provided standard rat chow and water ad libitum. All procedures with animals were carried out by the Federal Regulations for the Use and Care of Animals (NOM-062-ZOO-1999) issued by the Ministry of Agriculture of Mexico and approved by the Institutional Committee on Bioethics and Biosafety of the Institute of Instituto de Investigaciones Químico Biológicas of the Universidad Michoacana de San Nicolás de Hidalgo (Code-08-2022).

### 4.2. Induction of Diabetes and Experimental Design

Upon arrival, rats were housed for two weeks to familiarize them with their surroundings. Following this period, experimental diabetes was induced with a single dose of STZ. After 12 h of fasting, the rats were induced by an intraperitoneal injection of STZ freshly dissolved in citrate buffer (0.1 M, pH 4.5; Sigma-Aldrich, St. Louis, MO, USA) at doses of 60 mg/kg body weight, and control rats (normoglycemic) were injected only with buffer citrate. Twenty-four hours after STZ injection, blood glucose levels were determined with an Accu Check^®^ Performa glucometer (Roche, Indianapolis, IN, USA). Only the rats that exhibited fasting blood glucose of ≥250 mg/dL were employed for this study. After diabetes confirmation, the animals were randomly divided into four groups (*n* = 8 per group): normoglycemic rats (C), normoglycemic rats treated with apocynin (A), non-treated diabetic rats (D), and diabetic rats treated with apocynin (DA). For eight weeks, apocynin was administered daily at 3 mg/kg of body weight (vehicle, 0.1% dimethyl sulfoxide), intraperitoneally. Variations in body weight and glucose were evaluated every week.

Once treatment was complete, the animals of all groups were fasted for 8 h and sacrificed by decapitation. Blood samples were collected immediately for lipid profile determination. Skeletal muscle samples from EDL and soleus were dissected from both the left and right hind limbs and weighed. They were then stored at room temperature for morphological analysis and at −80 °C until processed by intramuscular triglycerides assay to measure lipid peroxidation levels and evaluate mRNA expression levels by RT-qPCR.

### 4.3. Lipid Profile

Serum was obtained by centrifuging blood samples at 3000× *g* for 5 min (Thermo Scientific Sorvall RC 6+ centrifuge, St. Louis, MO, USA). The Fujifilm NX500i system (Tokyo, Japan) was then used to determine levels of TG, total cholesterol, and very high-density lipoproteins (HDL-c) in serum. Low-density lipoproteins (LDL-c) and very low-density lipoprotein (VLDL-c) were calculated using the following formulas: VLDL-c = triglycerides/5, LDL-c = total cholesterol-(HDL-c + VLDL-c) [79].

### 4.4. Muscle Triglycerides Content

Muscle triglycerides from homogenate samples (EDL and Soleus) were obtained by the Aguilera-Méndez and Fernández-Mejía method with some modifications [80]. Briefly, 20 µg/µL of protein was resuspended in 1 mL of a solution containing 5% Triton-X100 in a PBS buffer. Samples were subjected to a water bath warming at 60 °C for 5 min and cooled slowly at room temperature. The samples were centrifuged at 13,300 rpm for 15 min. Triglycerides concentrations were determined from the supernatant with the reagent kit Spin react (BSIS49-E, Sant Esteve De Bas, Spain).

### 4.5. Muscle Histological Analysis

Skeletal muscle samples from the extensor digitorum longus (EDL) and soleus were fixed in 10% paraformaldehyde and subsequently embedded in paraffin. Transverse sections of 5 μm thickness were obtained using a microtome (Leica, Wetzlar, Germany) and stained with hematoxylin–eosin (HE) for histological analysis [81]. Twelve transverse sections per animal were randomly selected for morphological evaluation along both muscles. Images were acquired using digital photomicrography at 400× magnification (Leica DM3000). Quantitative analysis of fiber number and CSA was performed manually using NIH ImageJ software (version 1.54). For each muscle sample, fiber counting was performed manually by selecting four representative quadrants within a defined area (for example, 0.31 mm^2^). This method allowed a standardized analysis and minimized potential bias due to variations in the size of the evaluated fields. Regarding the spaces between muscle fibers, as shown in this study, they can vary due to multiple factors. In this case, the variation is primarily attributed to the pathophysiological condition of the tissue, which explains the differences observed among the experimental groups. However, care was taken to select fields with a homogeneous fiber distribution and without evident signs of artifacts, necrosis, or disproportionate fibrosis. The analyzed fields were chosen based on transverse sections in which the muscle fibers exhibited a regular circular or oval shape. This ensured appropriate and reliable comparison between samples.

### 4.6. Determination of Lipid Peroxidation

RNA Isolation According to our group’s method, a thiobarbituric acid reactive substances (TBARS) assay was used as a marker of lipid peroxidation in the EDL and soleus muscles [82].

### 4.7. RNA Isolation and RT-qPCR for mRNA Expression Analyses

Total RNA was isolated from muscle samples (EDL and soleus) to the TRIzol isolation reagent protocol (TRI Reagent, Sigma Aldrich, Saint Louis, MO, USA), described by Chomczynski and Sacchi [83] with minor modifications. RNA quality and quantity were analyzed spectrophotometrically at optical densities at 260/280 ratio using the BioPhotometer (Eppendorf, Hamburg, Germany). According to the manufacturer’s instructions, complementary DNA (cDNA) was synthesized from 2 μg of RNA using a cDNA synthesis kit (QIAGEN, Hilden, Germany). The Quantitative Reverse Transcription Polymerase Chain Reaction (qRT-PCR) assay was performed according to the method described by our group [28]. The sequences of the PCR primers used are shown in Table 1. The target gene expression was evaluated by the relative quantification method using the comparative delta–delta cycle threshold (ΔΔCT) method [84] with the endogenous housekeeping gene 18s as an internal control.

### 4.8. Statistical Analysis

All statistical tests used GraphPad Prism™ software version 10.3.1 (GraphPad Software Inc., San Diego, CA, USA). One-way analysis of variance (ANOVA) followed by Tukey’s multiple comparison test was used to analyze the data. The results were expressed as mean ± standard error of the mean (SEM), and the level of statistical significance was set at * *p* < 0.05.

## 5. Conclusions

In summary, this research has improved our understanding of the therapeutic potential of apocynin in the treatment of diabetic myopathy, which may be mediated by its anti-diabetic, hypolipidemic, and antioxidant properties. The findings imply that NOX en-zymes may play a role in the intramyocellular lipid buildup and diabetic muscle atro-phy in the fast and slow muscles of diabetic rats. This study indicates that apocynin mitigated diabetic muscle atrophy by decreasing NOX2-induced oxidative stress and triglycerides accumulation in muscles. Furthermore, it regulated the transcriptional expression of essential mediators of muscle atrophy and hypertrophy, such as atrogenes, *myostatin*, *follistatin*, and *PGC-1α*.

## Figures and Tables

**Table 1:** PCR primer sequences.

Gene	Primer Sequences
*MuRF-1*	Forward: 5′-GAAGTGATCATGGACCGGCA-3′
	Reverse: 5′-ACAAGGAGCAAGTAGGCACC-3′
*Atrogin-1*	Forward: 5′-AGCTTGTGCGATGTTACCCA-3′
	Reverse: 5′-GGTGAAAGTGAGACGGAGCA-3′
*Myostatin*	Forward: 5′-ATCACGCTACCACGGAAACA-3′
	Reverse: 5′-AGCTGGGCCTTTACCACTTT-3′
*Follistatin*	Forward: 5′-TGTGAAGACATCCAGTGCGG-3′
	Reverse: 5′-TCCGAGATGGAGTTGCAAGA-3′
*PGC-1α*	Forward: 5′-GAGGACACGAGGAAAGGAAGACT-3′
	Reverse: 5′-ACTGGCTTGAATCTGTGGAAGAAC-3′
*p47phox*	Forward: 5′-CTTCATTCGCCACATCGCCCTCC-3′
	Reverse: 5′-CACCTTCTCCGACAGGTCCTGCC-3′
*18s*	Forward: 5′-GCAAATTACCCACTCCCGAC-3′
	Reverse: 5′-CCGCTCCCAAGA TCCAACTA-3′

## Data Availability

The data used to support the findings of this study are included in the manuscript.

## Abbreviations

The following abbreviations are used in this manuscript:

IMCL	Intramyocellular lipids
PGC-1α	Peroxisome Proliferator-Activated Receptor Gamma Coactivator 1-alpha
DAGs	Diacylglycerols
ROS	Reactive oxygen species
EDL	Extensor digitorum longus
STZ	Streptozotocin
FA	Fatty acids
TG	Triglycerides
NOX	NADPH oxidase
SOD	superoxide dismutase
CSA	cross-sectional area
TBARS	Thiobarbituric acid reactive substances
COPD	chronic obstructive pulmonary disease
NF-κB	Nuclear factor-kappa B
TGF-β	Transforming growth factor-beta
p47phox	Neutrophil cytosolic factor 1
cDNA	complementary DNA
qRT-PCR	Quantitative reverse transcription polymerase chain reaction
